# Long-term effects of COVID-19 on the endocrine system – a pilot case-control study

**DOI:** 10.3389/fendo.2023.1192174

**Published:** 2023-09-18

**Authors:** Łukasz Szczerbiński, Michał Andrzej Okruszko, Maciej Szabłowski, Sebastian Sołomacha, Paweł Sowa, Łukasz Kiszkiel, Joanna Gościk, Adam Jacek Krętowski, Anna Moniuszko-Malinowska, Karol Kamiński

**Affiliations:** ^1^ Clinical Research Centre, Medical University of Bialystok, Bialystok, Poland; ^2^ Department of Endocrinology, Diabetology and Internal Diseases, Medical University of Bialystok, Bialystok, Poland; ^3^ Center for Genomic Medicine, Massachusetts General Hospital, Boston, MA, United States; ^4^ Programs in Metabolism and Medical and Population Genetics, Broad Institute of Massachusetts Institute of Technology (MIT) and Harvard, Cambridge, MA, United States; ^5^ Doctoral School at the Medical University of Bialystok, Bialystok, Poland; ^6^ Department of Population Medicine and Lifestyle Diseases Prevention, Medical University of Bialystok, Białystok, Poland; ^7^ Society and Cognition Unit, University of Bialystok, Bialystok, Poland; ^8^ Department of Infectious Diseases and Neuroinfections, Medical University of Bialystok, Białystok, Poland; ^9^ Department of Cardiology, University Hospital of Bialystok, Białystok, Poland

**Keywords:** COVID-19, SARS-CoV-2, thyroid hormones, endocrine system, obesity, diabetes

## Abstract

**Background:**

Coronavirus disease 2019 (COVID-19) has permanently changed the world. Despite having been a pandemic for nearly 3 years, the mid- and long-term complications of this disease, including endocrine disorders, remain unclear. Our study aimed to evaluate the lasting effects of COVID-19 on the endocrine system 6 months after initial infection.

**Methods:**

We compared patients who underwent COVID-19 to age- and sex-matched subjects from a population-based study conducted before the pandemic. We evaluated differences in multiple parameters related to metabolism and the endocrine system including fasting glucose, insulin, lipids, body composition, thyroid stimulating hormone (TSH), free thyroxine (fT4), free triiodothyronine (fT3), anti-thyroglobulin (aTG) and anti-thyroid peroxidase (aTPO) antibodies, prolactin, cortisol, testosterone, and estradiol.

**Results:**

We found significantly lower levels of fT3 and fT4, accompanied by higher levels of TSH and aTPO antibodies, in COVID-19 survivors. Moreover, we found that patients who underwent SARS-CoV2 infection had higher levels of prolactin and lower levels of testosterone than controls. Interestingly, differences in testosterone levels were observed only in male subjects. We did not detect significant differences in body composition or metabolic and glycemic parameters between cases and controls, except for significantly higher values of the HOMA2-B index in COVID-19 survivors.

**Conclusion:**

Our study indicates that severe acute respiratory syndrome coronavirus 2 (SARS-CoV-2) infection might have long-term consequences on the endocrine system, including the suppressed function of the thyroid gland, prolactin, and male sex hormone secretion. Moreover, we showed that in a 6-month follow-up, COVID-19 had no consequences on glycemic parameters, lipid profiles, liver function, body composition, cortisol levels, and estradiol levels.

## 1 Introduction

The coronavirus disease 2019 (COVID-19) pandemic, caused by severe acute respiratory syndrome coronavirus 2 (SARS-CoV-2), has significantly affected global healthcare systems. Although COVID-19 was initially identified as a respiratory infection, the disease can be characterized by multiple extrapulmonary manifestations affecting various tissues and organs, including the endocrine system. The variety of manifestations of viral infection is mainly driven by the widespread presence of the angiotensin-converting enzyme 2 (ACE2) receptor in the human body. ACE2 receptor is postulated to be a cellular gateway for SARS-CoV-2. Binding to the ACE2 receptor, a process that also requires the presence of transmembrane serine protease 2 (TMPRSS2) for viral spike glycoprotein protein priming, is an obligatory part of the SARS-CoV-2 cellular entry mechanism (1). ACE2 receptor and TMPRSS2 expression has been reported in several endocrine tissues including the pancreas, thyroid gland, ovaries, testes, and hypothalamus (1). This makes the endocrine system vulnerable to the damage induced by SARS-CoV-2 infection. Moreover, the pandemic has led to changes in dietary habits, often skewing towards unhealthier choices (2, 3), which could have long-term consequences on endocrine health.

The role of the endocrine system in SARS-CoV-2 infection is of particular interest, since endocrinopathies, such as obesity and diabetes, have been recognized as one of the strongest risk factors for the development of severe COVID-19 (4–9). Finally, the potential mechanisms of virus-induced endocrine complications of COVID-19, including direct viral injury, endothelial dysfunction in the highly vascular structures of endocrine glands, cytokine-induced injury, or dysregulation of the renin-angiotensin-aldosterone system (RAAS), might be accompanied by side-effects of the drugs used in COVID-19 management (steroids and antiviral agents) (1, 10, 11).

The effects of COVID-19 are not limited to the acute phase of the disease and predominant respiratory symptoms. Growing evidence shows that a significant number of people present with persistent symptoms for weeks or even months after initial infection. These long-term effects of COVID-19 are referred to as long COVID, post-acute sequelae of SARS-CoV-2 (PASC), or post-COVID syndrome. They may include fatigue, shortness of breath, cognitive problems, headache, sleep problems, depression, anxiety, joint and muscle pain, hair loss, or reduced libido (12). Most of these symptoms overlap with the clinical presentation of selected endocrinopathies, including thyroid or adrenal gland dysfunction. Thus, an understanding of how COVID-19 affects the endocrine system is of particular interest, as proper diagnosis and management of these endocrinopathies may resolve the persistent symptoms experienced by patients in the long term after SARS-CoV-2 infection. There are existing reports showing alterations in the function of selected endocrine glands, including reports of new-onset diabetes, subacute thyroiditis, euthyroid sick syndrome, and pituitary apoplexy (13); however, knowledge of the long-term effects of COVID-19 on the endocrine system is still very limited.

Thus, our study aimed to evaluate the long-term effects of SARS-CoV-2 infection on the endocrine system by comparing the hormonal activity of multiple glands in two distinct groups of patients. The first group consists of patients who underwent COVID-19 and were examined six months after infection. The second group, serving as a control, consists of age- and sex-matched subjects from a population study examined before the COVID-19 pandemic.

## 2 Materials and methods

### 2.1 Study design

In this study, we included two groups, cases and controls, to evaluate the effects of COVID-19 on endocrine system function. The case group was composed of adult subjects with a history of SARS-CoV2 infection (confirmed by a polymerase chain reaction test) and hospitalization due to COVID-19 who participated in the “Rise or fall? Short- and long-term health and psychosocial trajectories of the COVID-19 pandemic” project conducted at the Medical University of Bialystok. For the present study we selected 39 patients (26 females and 13 males) that were examined approximately 6 months after infection and did not report any endocrine disorders diagnosed before COVID-19 infection. The control group was composed of participants from the ongoing Bialystok PLUS (Polish Longitudinal University Study) cohort, which provides broad information on the health of Bialystok (Poland) residents. For the purpose of the study we selected 39 (26 females and 13 males) age-, sex-, and Body mass index (BMI)-matched case group subjects, without diagnosed endocrine diseases, from the Bialystok PLUS cohort that were examined between 2017-2020 (pre-COVID-19). Both case and control group subjects participated in a single visit, where phenotypic assessments, including hormonal status, were performed in the fasting state. All participants provided informed consent prior to participating in the project. The study was conducted in accordance with the Helsinki Declaration and adhered to Good Clinical Practice guidelines. The study protocols were approved by the Bioethics Committee of the Medical University of Bialystok (approval numbers: APK.002.346.2020 and R-I-002/108/2016).

### 2.2 Laboratory analyses and body composition measurements

The laboratory analyses included in this study were performed using fasting venous blood samples, collected in the morning, after an overnight fast of approximately 10 hours, typically between 07:30 and 08:30 h. All parameters were assessed through a single measurement. Thyroid-stimulating hormone (TSH), free triiodothyronine (fT3), free thyroxine (fT4), anti-thyroid peroxidase antibodies (aTPO), anti-thyroglobulin antibodies (aTG), insulin, prolactin, cortisol, testosterone, and dehydroepiandrosterone sulfate (DHEA-S) concentrations were measured using an electrochemiluminescence method on a Cobas e411 (Roche Diagnostics, Switzerland). Biochemical measurements of serum triglyceride (TG), total cholesterol (TChol), high-density lipoprotein cholesterol (HDL), aspartate aminotransferase (AST), alanine aminotransferase (ALT), and low-density lipoprotein cholesterol (LDL) concentrations were performed using colorimetric methods with Cobas c111 (Roche Diagnostics, Switzerland). Hemoglobin A1c (HbA1c) was measured using high-performance liquid chromatography (HPLC) (Bio-Rad VARIANT, Bio-Rad Laboratories, USA). Anthropometric measurements were performed using a calibrated stadiometer SECA 264 (SECA, Hamburg, Germany) and electronic scale (SECA 769, SECA, Hamburg, Germany). BMI was calculated as body mass (kg) divided by height (m) squared. Whole-body dual-energy X-ray absorptiometry (DXA) was performed for body composition analysis using Lunar iDXA (GE Healthcare, Chicago, USA). Total lean body mass, fat mass, bone mass, and visceral adipose tissue (VAT) mass were also measured.

### 2.3 Statistical analyses

The clinical and demographic parameters were summarized using the number of observations, arithmetic mean, median, and standard error. We used Student’s t-test to compare normally distributed data, and the Mann-Whitney-U test for non-normally distributed data. The difference in sex structure between the groups was checked using the chi-squared test.

We evaluated the impact of sex on the studied parameters through a two-way ANOVA, with sex as predictor and case/control as response. Correlations between hormones were estimated with Pearson’s correlations and corresponding p-values. To account for multiple comparisons, the p-values were corrected using with the Benjamini–Hochberg procedure and we reported resulting False Discovery Rates (FDRs). Statistical significance was set at p < 0.05. R software v3.5 (14) was used to perform the analyses and generate figures.

In our pilot study, we did not perform a power analysis prior to initiating the study due to the difficulty of recruiting patients for this data with the goal to maximize sample size, but instead estimated the power after collecting the data. The sample size was sufficient to determine the existence of statistically significant differences between the groups, as indicated by the calculated Cohen’s d effect size and significant corrected p-values.

## 3 Results

The clinical characteristics of the study groups are presented in **Table 1**. In line with the study design, there were no statistically significant differences in sex distribution, age, or BMI between the groups.

**Table 1:** Clinical characteristics of studied groups.

Feature (unit)	Cases			Controls			p-value
	Mean	Median	SE	Mean	Median	SE	
**Age (years)**	48.64	47.00	2.24	48.79	47.00	2.22	0.960
**BMI (kg/m^2^)**	28.60	27.80	0.89	28.40	28.30	0.79	0.880
**Total fat mass (kg)**	29.78	28.68	1.76	29.64	29.39	1.45	0.684
**Total lean mass (kg)**	48.46	43.80	1.72	48.31	44.56	1.82	0.921
**Total bone mass (kg)**	2.60	2.62	0.08	2.64	2.61	0.09	0.763
**VAT mass (kg)**	1.47	0.96	0.18	1.41	1.15	0.02	0.889
**Fasting glucose (mg/dl)**	99.69	99.00	1.90	103.23	97.00	4.33	0.940
**HbA1c (%)**	5.41	5.40	0.08	5.41	5.40	0.10	0.840
**Fasting insulin (μU/mL)**	17.70	14.65	2.84	13.22	10.72	1.14	0.204
**HOMA2-IR**	2.28	1.90	0.34	1.75	1.40	0.15	0.240
**HOMA2-B%**	126.78	123.60	8.29	107.19	102.80	6.04	**0.047**
**Total cholesterol (mg/dl)**	188.69	186.00	5.95	192.56	191.00	6.28	0.660
**Triglycerides (mg/dl)**	129.56	102.00	14.95	104.51	87.00	6.99	0.410
**HDL-cholesterol (mg/dl)**	61.73	58.82	2.68	60.40	59.71	2.21	0.700
**LDL-cholesterol (mg/dl)**	119.61	120.80	5.80	129.76	129.50	5.32	0.200
**AST (U/L)**	22.03	20.10	0.99	23.36	20.90	2.01	0.960
**ALT (U/L)**	25.21	18.4	3.01	25.36	18.90	4.77	0.980
**TSH (μIU/mL)**	2.11	1.94	0.19	1.420	1.330	0.12	**0.003**
**fT3 (pmol/L)**	4.96	4.92	0.07	5.33	5.240	0.08	**0.002**
**fT4 (pmol/L)**	15.51	16.06	0.30	17.56	17.29	0.44	**0.000**
**aTG (IU/mL)**	67.48	17.12	19.42	65.08	19.65	27.03	0.681
**aTPO (IU/mL)**	68.78	13.48	22.72	16.97	10.34	2.44	**0.040**
**prolactin (μIU/mL)**	294.07	229.70	23.65	227.39	179.70	19.72	**0.016**
**Cortisol (μg/dL)**	12.51	11.33	0.88	12.93	11.89	0.73	0.426
**Testosterone (ng/mL)**	1.14	0.25	0.24	1.56	0.26	0.32	0.196
**DHEA-S (μg/dL)**	176.94	182.40	13.03	201.74	193.30	16.32	0.239
**Estradiol (pg/mL)**	61.90	28.80	15.20	63.29	34.63	14.43	0.779
**SHBG (nmol/L)**	79.82	64.67	9.97	55.59	53.51	3.39	0.137

BMI, Body Mass Index; VAT, visceral adipose tissue; HOMA2, Homeostasis Model Assessment 2 (IR - estimated insulin resistance, B% - estimated beta cell function); HDL-cholesterol, High-Density Lipoprotein Cholesterol concentration; LDL - cholesterol, High-Density Lipoprotein Cholesterol concentration; AST, aspartate aminotransferase concentration; ALT, alanine aminotransferase concentration; TSH, thyroid stimulating hormone concentration; fT3, free triiodothyronine concentration; fT4, free thyroxine concentration; aTG, anti-thyroglobulin antibodies concentration; aTPO, anti-thyroid peroxidase antibodies concentration; DHEA-S, dehydroepiandrosterone sulphate concentration; SHBG, sex hormone binding globulin; SE, standard error.

bold values in the p-value column represent statistically significant p-value (p<0.05).

### 3.1 Glycemic, metabolic and body composition parameters

In the comparisons between the control and case groups, we did not find significant differences in glycemic or metabolic parameters, including HbA1c, fasting glucose, insulin, lipids, and liver transaminase concentrations. However, we found that the case group presented significantly higher values of the HOMA2-B index (p=0.047), with a lack of differences in the HOMA-IR index (see **Table 1**). Moreover, we did not find any significant differences between the case and control groups in any of the measured body composition parameters, including total fat mass, lean mass, bone mass, and VAT mass.

### 3.2 Thyroid function

**Figure 1:**
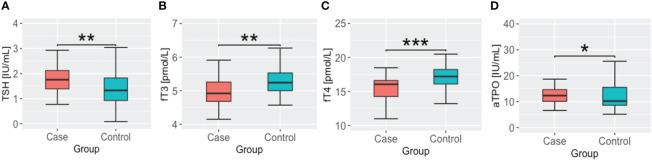
TSH, thyroid hormone and antithyroid antibody concentrations in cases and controls groups. **(A)** thyroid stimulating hormone – TSH; **(B)** free triiodothyronine – fT3; **(C)** free thyroxine – fT4; **(D)** anti thyroid peroxidase antibody – aTPO; * - p <.05; ** - p<.01; *** - p <.001.

We found significantly lower fT3 and fT4 concentrations in the case group compared to the controls (p=0.002), accompanied by higher TSH concentrations (p=0.003) (see **Figure 1**). Moreover, we found that patients in the case group had higher concentrations of aTPO (p=0.04), with no significant difference in anti-TG antibody levels between the groups. The two-way ANOVA analysis did not show significant interactions between sex and case/control status in thyroid function parameters.

### 3.3 Other hormones

**Figure 2:**
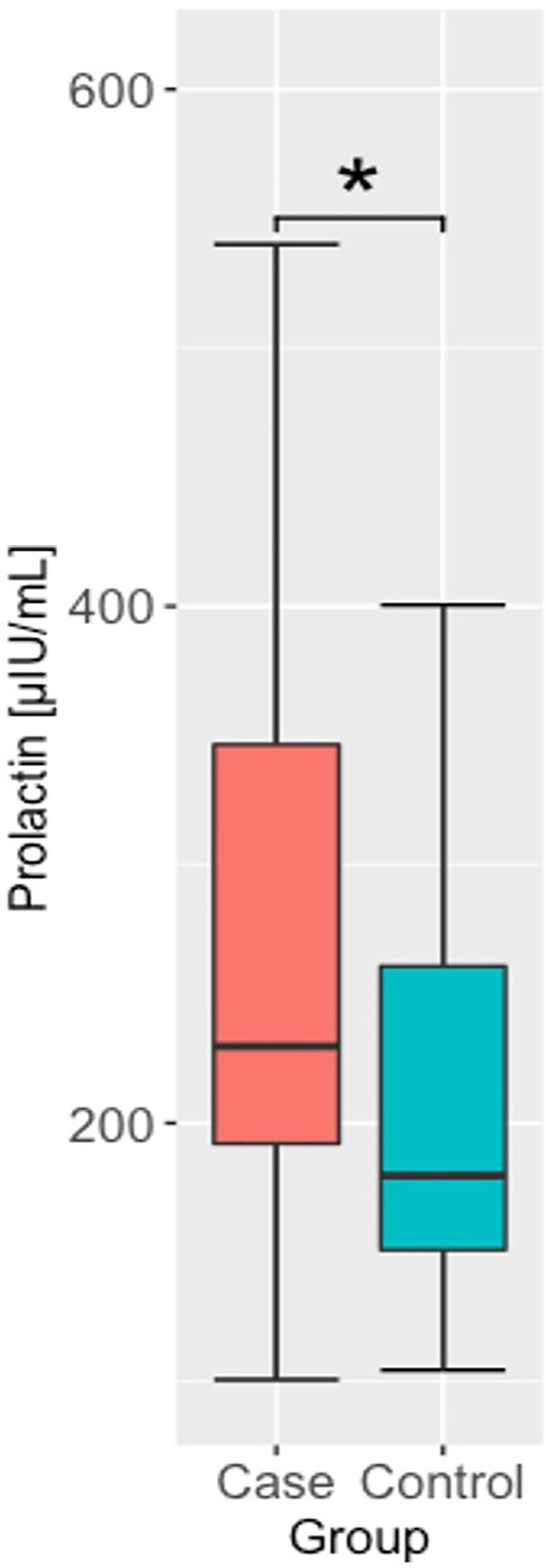
Prolactin concentrations in cases and controls groups. * - p <.05.

**Figure 3:**
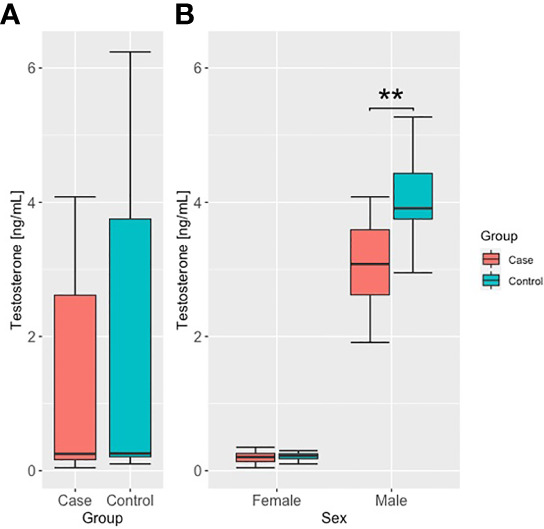
Testosterone concentrations in case and control groups, **(A)** unstratified, and **(B)** stratified by sex. ** - p<.01.

Among the other hormones studied, we found significant differences in prolactin levels between the case and control groups, with the case group having higher prolactin levels than the controls, as shown in **Figure 2**. There were no significant interactions between sex and case/control status. Given the relationship between prolactin secretion and the function of the thyroid gland, we also evaluated the correlation between prolactin and TSH levels and thyroid hormones. However, we did not observe any significant correlations. Finally, we found a significant difference in testosterone levels, with lower values in the case group (p=0.009) (see **Figure 3**). The interaction analysis revealed that this is only specific to male subjects. We found no differences in DHEA-S, estradiol, SHBG, and cortisol levels between the studied groups.

## 4 Discussion

In the past two years, our understanding of the biology of COVID-19 and its long-term complications has drastically improved. However, the effects of this disease on the endocrine system remain unclear. There are multiple case reports on acute complications related to different endocrine glands; however, there are very limited data on the long-term impacts of the infection on hormonal homeostasis. In our study, we comprehensively and simultaneously assessed the hormonal functions of different parts of the endocrine system 6 months after initial COVID-19 infection. We found that patients who underwent the disease presented significant differences in thyroid function and testosterone and prolactin secretion, with no significant impact on the glycemic and metabolic parameters, cortisol, or estradiol concentrations. Detected hormonal differences in COVID-19 survivors might explain, at least partially, the common symptoms described as long-COVID, including general fatigue and depression. Notably, the identified differences can be eminently treatable, and correct diagnosis and management of observed dysregulated hormones can result in the improvement of symptoms observed in patients with long-COVID.

The relationship between impaired glycemia, metabolic syndrome, and COVID-19 susceptibility is well established (15); however, the metabolic outcomes of SARS-CoV2 infection remain unclear. A growing number of studies have indicated that infection can lead to insulin resistance, hyperglycemia, and even diabetes in both normal and overweight subjects (16, 17). For example, Chen et al. (18) showed that COVID-19 may increase the risk of insulin resistance in patients without diabetes. This can be a consequence of several factors, including virally-induced inflammation and immune dysfunction, or the side effects of glucocorticosteroid treatment for COVID-19. Moreover, Chen et al. found that at 3- and 6-month follow-ups after infection, fasting levels of C-peptide and HOMA for beta cell function were significantly increased (18). In our study we showed significantly higher HOMA-B% values in subjects who recovered from COVID-19 than in the control group, indicating an increased secretory activity of pancreatic islet beta cells. It can be speculated that this is a compensatory response mechanism to the initial stage of insulin resistance, when increasing insulin levels are required to maintain glucose homeostasis. In contrast, our study did not indicate any other metabolic consequences of SARS-CoV2 infection such as hyperglycemia, dyslipidemia, or changes in body composition and weight. However, it is important to note that, in our study, we used HOMA indices, which may not fully reflect the secretion/clearance balance of insulin. To better understand the consequences of COVID-19 infection on insulin resistance and insulin secretion, further studies using clamp techniques are needed (19).

Abnormal thyroid function is a frequently reported complication of COVID-19 - particularly since most of the “long COVID” symptoms, like fatigue, brain fog, or myalgia, share many similarities with thyroid dysfunction. Several reports of subacute thyroiditis, autoimmune thyroiditis, and hypothalamic-pituitary-thyroid axis dysfunction after SARS-CoV2 infection have been published. Unfortunately, these studies were based on small groups and relatively short intervals between infection resolution and analysis (20). Our study found significantly higher TSH levels and lower fT3 and fT4 levels in patients who recovered from COVID-19. Moreover, the post-COVID group had significantly higher concentrations of aTPO antibodies. This indicates that autoimmune hypothyroidism is a potential complication of COVID-19. However, it is important to note that in our study, concentrations of thyroid function parameters did not meet the thresholds for diagnosis of hypothyroidism. Long-term studies evaluating thyroid function after COVID-19 that could be used to create recommendations for management after infection are lacking (21). Our study suggests that monitoring thyroid function after COVID-19 is of particular importance in evaluating whether thyroid function returns to baseline or progresses to overt thyroid disease.

Previous studies have reported that reduced testosterone levels are associated with the course of COVID-19, including severe outcomes (22). Moreover, males infected with SARS-Cov-2 were characterized by reduced testosterone levels, potentially due to direct damage to the Leydig cells, which were observed during the acute phase of COVID-19, with progressive improvements throughout the recovery phase (21, 23). This is in line with our findings, where significantly lower levels of circulating testosterone were found in males who underwent COVID-19.

Finally, we found that in the group of COVID-19 survivors, prolactin concentrations were significantly higher compared to the control group. It is well established that the elevation of prolactin levels is a form of response to various stresses, including infections (24, 25). The results of experimental research proved that prolactin acts as an anti-inflammatory and immunomodulatory factor (26) and thus may play an important role in limiting COVID-19 hyperinflammation. Data on prolactin levels after COVID-19 are very limited, with only a few studies reporting increased hormone concentrations during follow-up (27, 28). Thyroid dysfunction could be a contributing factor to elevated prolactin levels, as it is known to affect prolactin secretion. However, our study results did not show significant correlations between prolactin and thyroid hormone levels, indicating that other mechanisms may be responsible for the increased prolactin levels seen in COVID-19 survivors.

Our study has several limitations, including the relatively small sample size and the case-control design. Moreover, it is important to note that the female participants’ menstrual cycle phase data were unavailable for this study. This factor could have a potential impact on both endocrine and metabolic data. Additionally, this study primarily serves as a screening of the endocrine system following SARS-CoV-2 infection rather than a detailed analysis of the hormonal function of each organ. Deeper analyses with more sophisticated measurements, broader coverage of hormones, and consideration of the chronobiology of their secretion would provide a more comprehensive understanding of the impact of COVID-19 on the endocrine system. Despite these limitations, we believe that the results from our pilot study provide valuable insights into the relationships between COVID-19 and endocrine system function. To fully understand the consequences of SARS-CoV-2 infection on the endocrine system, further studies are needed with increased sample sizes, more comprehensive endocrine system assessments (including functional tests and evaluations of the hypothalamic-pituitary axis), and preferably with an observational design where clinical assessment can be performed in each subject before and after infection.

## 5 Conclusions

In our study, we showed that SARS-CoV-2 infection may have long-term consequences on the endocrine system. Our results indicate that patients who underwent COVID-19 present with suppressed thyroid gland function in a potentially autoimmune mechanism. Moreover, infection affects prolactin secretion and male sex hormone levels. We also showed that in a 6-month follow-up, COVID-19 had no lasting effects on glycemic parameters, lipid profiles, liver function, body composition, cortisol levels, or estradiol levels. These findings have important implications in the clinical assessment of post-COVID assessment of patients, as with the growing number of COVID survivors, this is an emerging issue in modern medicine.

## Data availability statement

The raw data supporting the conclusions of this article will be made available by the authors, without undue reservation.

## Ethics statement

The studies involving humans were approved by Bioethics Committee at the Medical University of Bialystok. The studies were conducted in accordance with the local legislation and institutional requirements. The participants provided their written informed consent to participate in this study.

## Author contributions

ŁS: made substantial contributions to the study conception and design, acquisition, analysis, and interpretation of data, and co-wrote the manuscript. MO, MS, and JG: prepared the figures, analyzed and interpreted the data, and co-wrote the manuscript. JW: analyzed and interpreted the data, and co-wrote the manuscript. SS, PS, and ŁK: made substantial contributions to the study conception and design, acquisition, analysis, and interpretation of data. AK and AM-M: was involved in revising and editing the manuscript. KK: was involved in the conception and design of the study, revising and editing the manuscript, funding acquisition, and project administration. All authors contributed to the discussions and read and approved the final manuscript.
